# Challenges in Interpreting Thyroid Stimulating Hormone Results in the Diagnosis of Thyroid Dysfunction

**DOI:** 10.1155/2019/4106816

**Published:** 2019-09-22

**Authors:** Salman Razvi, Sindeep Bhana, Sanaa Mrabeti

**Affiliations:** ^1^Institute of Genetic Medicine, Newcastle University, Newcastle-upon-Tyne NE1 3BZ, UK; ^2^Department of Internal Medicine, Division of Endocrinology and Diabetes, University of the Witwatersrand, Johannesburg, South Africa; ^3^Medical Affairs EMEA, Merck Serono Middle East FZ-LLC, Dubai, UAE

## Abstract

The pituitary hormone, thyrotropin (TSH), is regarded as the primary biomarker for evaluating thyroid function and is useful in guiding treatment with levothyroxine for patients with hypothyroidism. The amplified response of TSH to slight changes in thyroid hormone levels provides a large and easily measured signal in the routine care setting. Laboratories provide reference ranges with upper and lower cutoffs for TSH to define normal thyroid function. The upper limit of the range, used to diagnose subclinical (mild) hypothyroidism, is itself a matter for debate, with authoritative guidelines recommending treatment to within the lower half of the range. Concomitant diseases, medications, supplements, age, gender, ethnicity, iodine status, time of day, time of year, autoantibodies, heterophilic antibodies, smoking, and other factors influence the level of TSH, or the performance of current TSH assays. The long-term prognostic implications of small deviations of TSH from the reference range are unclear. Correction of TSH to within the reference range does not always bring thyroid and other biomarkers into range and will not always resolve the patient's symptoms. Overt hypothyroidism requires intervention with levothyroxine. It remains important that physicians managing a patient with symptoms suggestive of thyroid disease consider all of the patient's relevant disease, lifestyle, and other factors before intervening on the basis of a marginally raised TSH level alone. Finally, these limitations of TSH testing mitigate against screening the population for the undoubtedly substantial prevalence of undiagnosed thyroid disease, until appropriately designed randomised trials have quantified the benefits and harms from this approach.

## 1. Introduction

The prevalence of treated hypothyroidism is increasing in both the United Kingdom and the United States [1, 2]. Furthermore, the global prevalence of undiagnosed thyroid dysfunction, at least in the developed world, is falling, probably due to a combination of iodine supplementation in iodine-deficient areas, widespread and frequent thyroid function assessment, and lower thresholds to commence treatment [3–5]. Approximately, 1–3% of the population has hypothyroidism in iodine-replete areas, with much higher prevalence in older persons and in women [5, 6].

The symptoms of thyroid dysfunction are often nonspecific, and diagnosis is confirmed by laboratory tests for thyroid hormones and the pituitary hormone thyrotropin (TSH). Current guidelines for the diagnosis and management of thyroid dysfunction focus primarily on the measurement of TSH, as the most sensitive and specific marker of systemic thyroid status, with test results interpreted according to defined reference ranges [7–9]. However, serum TSH has several limitations, and “normal” levels are not necessarily indicative of tissue-specific thyroid hormone status. The purpose of our review is to summarise the evidence-based rationale for current thyroid testing practices and to address common pitfalls in the interpretation of challenging results. Furthermore, it is important to remember that TSH is a pituitary hormone and ensuring normal pituitary function is vital prior to interpreting its circulating levels and its relationship with thyroid hormones.

## 2. Overview of TSH and Relationship with Thyroid Hormones

The physiology of thyroid hormone regulation has been reviewed extensively elsewhere [10, 11]. Accordingly, only a brief account will be given here, focussing on those aspects of the hypothalamus-pituitary-thyroid axis that are most relevant to the design and use of thyroid function tests. The regulation of thyroid hormone secretion is conducted from within the central nervous system, which allows modulation of the system from periphery, via feedback from nutrient intake or via the autonomic nervous system [11]. In brief, thyrotropin-releasing hormone (TRH) is secreted from the hypothalamus and reaches the anterior pituitary via the hypophyseal portal circulation. Activation of TRH receptors stimulates the release of TSH, which activates its own receptors on the follicular cells of the thyroid gland. This causes increased cellular uptake of iodine from the blood, increased synthesis of thyroglobulin, and secretion into the blood stream of triiodothyronine (T3) and thyroxine (T4) via activation of the enzyme thyroid peroxidase (TPO).

Feedback circuits result in an inverse relationship between serum levels of thyroid hormones and TSH, i.e., low T4 (as observed in hypothyroidism) and high T4 (as seen in hyperthyroidism) levels are associated with elevated and low TSH, respectively. The relationship between the magnitude of changes in serum TSH and the resulting magnitude of changes in circulating thyroid hormones is the key aspect of the regulation of thyroid function with regard to diagnosis of thyroid disorders. The precise nature of the relationship remains under debate, but the relationship between TSH and T4 approximates to an inverse log-linear relationship for most individuals, and this relationship becomes clearer for a given individual when more data points are available to define it [12–14].

Importantly, a halving of circulating T4 is accompanied by up to a 100-fold increase in serum TSH. Furthermore, the relationship between TSH and T4 varies amongst individuals and is affected, amongst other factors, by age, smoking, levothyroxine treatment, and the presence of antibodies [12, 15, 16]. Clearly, these large alterations in TSH are more amenable to identification by routine measurements in clinical laboratories than small variations in T4, and this explains the use of TSH measurements as the gold standard for the diagnosis of thyroid disorders in current guidelines for the management of hypothyroidism [7, 9].

## 3. Serum TSH as the Principal Diagnostic Marker of Systemic Thyroid Status

### 3.1. Evolution of the TSH Test

Prior to the development of TSH and thyroid hormone testing in serum, other biological markers of thyroid hormone status such as basal metabolic rate were used to diagnose thyroid dysfunction and also to titrate thyroid hormone replacement doses [17]. Testing for thyroid function began in the 1950s with the development of an indirect test for total T4 [18]. Since then, continual development of radioimmuno- and liquid chromatography-tandem mass spectrometry-based assay technology has led to the availability to physicians in the routine care setting of accurate and precise measurement of TSH, T4, T3, thyroid hormone-binding proteins, thyroglobulin, and various autoantibodies of diagnostic interest [18]. Quantitative measurements of serum TSH by radioimmunoassay were first reported by Utiger [19]. The early TSH assays had a limit of detection of approximately 1 mIU/L, which was sufficient to diagnose primary hypothyroidism [7, 9]. Increases in the sensitivity of TSH tests were driven by the need to quantify much lower TSH levels associated with the diagnosis of hyperthyroidism, particularly the subclinical form.

Interpretation of a biomarker test result depends on the ability to distinguish normal from abnormal. The population reference range for “normal” TSH is defined as containing 95% of a “normal” population who are believed to be free of conditions that could influence TSH levels, with 2.5% of subjects above and below the range [7]. Reference ranges may be device-, laboratory-, and population-specific, and “normal” or “abnormal” results may be diagnosed using reference ranges from populations local to the laboratory [20].

Guidelines cite a reference and therapeutic target range for TSH to define normal thyroid function in adults at 0.4–4.0 mIU/mL [9]. Subclinical hypothyroidism is defined as TSH above the reference and when thyroid hormone levels are normal; conversely, subclinical hyperthyroidism is characterised by T4 and T3 within the normal range and low TSH. In most laboratories, measurement of FT4 only occurs when TSH is out of range. A large observational study from Australia showed that restricting free T4 (FT4) measurements to patients whose serum TSH was clearly outside its reference range (<0.2 mIU/L or >6 mIU/L) had little or no impact on the diagnostic utility of the TSH test [21].

The management of overt thyroid dysfunction is not disputed, and there is widespread agreement that this condition should be treated [8, 9]. The question remains as to how to proceed in the case of a borderline TSH test. Current European guidelines recommend a trial of levothyroxine treatment for younger patients (<65–70 y) with symptoms reminiscent of hypothyroidism and mildly elevated TSH (<10 mU/L) [22]. Larger elevations of TSH in these younger patients require treatment with levothyroxine irrespective of the presence or absence of symptoms [22]. A strategy of watchful waiting is appropriate for very elderly patients, with use of levothyroxine where clearly necessary [22].

### 3.2. Limitations of TSH as a Diagnostic Marker of Thyroid Function

**Figure 1:**
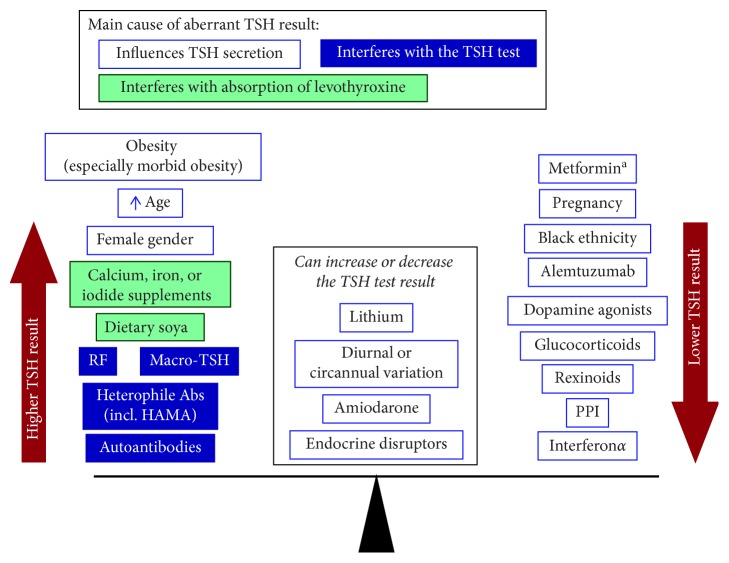
Overview of common factors that can produce an aberrant TSH test. ^a^Metformin reduces serum TSH in people with overt or subclinical hypothyroidism and in euthyroid individuals with high normal baseline TSH levels (see refs [31] and [32]; see text for other references). HAMA: human anti-mouse antibody; RF: rheumatoid factor; TSH: thyrotropin.

The introduction of cutoff values for TSH renders the diagnosis of thyroid disease categorical, in that even a small increase in TSH can in principle lead to the patient potentially acquiring the lifelong label of a medical condition. A number of demographic, diagnostic, and disease-related factors, described briefly below, influence the TSH level and should be considered when making the diagnosis of hypothyroidism (Figure 1).

#### 3.2.1. Age and Gender

A study in a population of about 150,000 thyroid antibody-negative people, without the history of treatment for a thyroid-related condition, found that the 97.5^th^ percentile for TSH (i.e., the upper limit for a TSH reference range in this population) increased from 3.98 mIU/L at age 31–40 to 5.94 mIU/L at age >90 years [20]. Increasing TSH with age was observed for both men and women in this study, while this and other studies have demonstrated higher levels of TSH in women than in men [14, 20, 23, 24]. A representative survey in Korea found the lowest TSH levels in middle-aged individuals, with higher values in younger and older age groups [23]. The effect of TSH on the rate of conversion of FT4 to FT3 is age-dependent, with increasing FT3/FT4 ratios seen as TSH increases, at least up to middle age [15]. The response of TSH levels to hypothyroidism may be larger in younger subjects [12]. These data support the use of locally derived, age-specific, and gender-specific reference ranges for TSH.

#### 3.2.2. Ethnicity

A study from a large, nationally representative, population-based cohort in the USA found higher levels of TSH in non-Hispanic white vs. non-Hispanic black or Mexican American subjects [24]. A high titre of thyroid autoantibodies is also less prevalent in black vs. white subjects in this study. Data from Brazil demonstrated a lower prevalence of hypothyroidism (but more hyperthyroidism) in black subjects vs. other ethnicities [25]. Ethnicity also influences thyroid hormone levels, which could produce an aberrant test where the local population that supplied subjects for determining the reference range is mainly of a different ethnicity to the patient [26].

#### 3.2.3. Medications and Supplements

Numerous concurrent medications and supplements can interfere with thyroid function tests (summarised in Figure 1) [27, 28]. Metformin, the most commonly prescribed pharmacologic treatment for type 2 diabetes, reduces TSH with no change in FT4 in treated or untreated hypothyroid patients [29] or in patients with benign thyroid nodules [30]. A meta-analysis showed that metformin treatment reduced TSH levels in patients with overt, levothyroxine-treated hypothyroid disease, or subjects with untreated subclinical hypothyroidism, but not in euthyroid individuals [31]. Another study, however, concluded that metformin treatment reduces serum TSH levels that are towards the upper limit of the reference range independent of anti-thyroid antibody status [32]. Amiodarone may induce a transient or sustained hypothyroid-like condition (increased TSH) or thyroiditis (decreased TSH). Alemtuzumab, proton pump inhibitors, antiepileptic drugs, glucocorticoids, rexinoids, dopamine agonists, somatostatin analogues, interferon-*α*, and tyrosine kinase inhibitors are among other drugs known to alter TSH levels [27]. Dietary iodide supplements increase the TSH activity, and variations in local iodide intake may affect TSH reference ranges [28]. Dietary soya and supplements, such as those containing calcium or iron, may interfere with the absorption of levothyroxine and thus increase TSH [33].

#### 3.2.4. Interference with Assays

Automated immunoassays are vulnerable to interference that can affect thyroid function results and thus have impact on clinical decision making. It is estimated that interference is prevalent in almost 1% of all thyroid function tests, and therefore, the scale of the problem is potentially enormous. Interference with thyroid function tests should be suspected when the result is discordant with the clinical or other biochemical findings [27]. People with hypothyroidism may produce a form of TSH, “macro-TSH,” made up of TSH coupled with IgG autoantibodies, which may be recognised to different extents by different commercial assays for TSH [34]. This may provide another potentially important source of both interpatient variation and discordance between the reported TSH level and the individual patient's clinical thyroid status.

Finally, a “sandwich” immunoassay-based TSH test may use non-human antibodies directed against TSH, where immobilised antibodies capture TSH in the sample, and antibodies directed against other TSH epitopes generate the assay signal. A patient's serum may contain heterophilic antibodies, which are antibodies directed against animal antibodies of the same species used in the assay. These human anti-animal antibodies may bridge between the capture and detection of antibodies in the assay and generate a false positive signal, leading to a falsely elevated TSH measurement [27, 35]. Human anti-mouse antibody (HAMA) is the most common heterophilic antibody, among others. Assays contain defences against heterophilic antibodies, although these may be overwhelmed if the interfering antibody is present at a high enough titre. Rheumatoid factors (RF), IgM species directed against human IgG, are present in patients with autoimmune diseases, particularly rheumatoid arthritis [35]. RF acts like HAMA by binding to antibodies used in the TSH assay and thus altering the test result. Biotin, antistreptavidin antibodies, antiruthenium antibodies, and thyroid hormone autoantibodies provide other sources of interference with the TSH test that may lead to an aberrant result [36].

#### 3.2.5. Individual “Set Points” for Thyroid Hormones and Risk of End-Organ Damage

Diagnostic reference ranges are, by definition, a one-size-fits-all solution to the challenge of interpreting biomarker results [37]. Longitudinal measurement of thyroid hormones in healthy individuals has shown that levels of T3, T4, and FT4 index vary little over time [38]. Each individual appears to have his/her own, unique relationship between TSH and T3 or T4, which is partly genetically determined [39] and influenced relatively little by biological variation [40]. By implication, TSH may reflect the characteristics of the individual's hypothalamic-pituitary-thyroid axis as much as it reflects a holistic estimate of metabolic function.

A TSH value slightly above or below the reference range (with other thyroid hormones within their ranges) might be appropriate for this individual but could nevertheless precipitate a diagnosis of subclinical thyroid disease. One proposal has been to define quantitatively each patient's unique euthyroid set point to guide management [41]. This approach may prove to be both cumbersome and expensive. Treatment with levothyroxine has been shown to influence an individual's set point for thyroid function, with higher FT4 and lower FT3, despite a lower TSH level compared with untreated patients [42]. An alternative approach could be to define optimal ranges for TSH and FT4 based on risk engine-derived estimates of the risk of adverse cardiovascular outcomes, in a manner analogous to that used for setting treatment goals in the management of serum lipids [43].

#### 3.2.6. Diurnal and Circannual Variations in TSH Levels

A diurnal variation exists for TSH levels in euthyroid and hypothyroid patients (treated or untreated), with lower values in the daytime [44, 45]. The magnitude of the circadian rhythm in TSH is greater for older people due to a larger increase in nocturnal TSH production, and the circadian rhythms for TSH have been observed to differ according to ethnicity [46]. The nadir in TSH levels occurs around the middle of the day [44] so that the daytime sampling of blood for TSH measurements should minimise the interference with the test from this source. A circannual variation for TSH has been reported with lower values recorded in the summer [47], although another study reported no change in the TSH reference range at different times of the year [46].

#### 3.2.7. Pregnancy

The size of the thyroid gland, thyroid hormone production, and iodine requirements all increase markedly during pregnancy, with a concomitant fall in the TSH level. Current guidelines for the management of thyroid disease accept the need for a lower upper limit of the reference range during different stages of pregnancy, but differences between populations in the magnitude of the effect of pregnancy on thyroid hormone levels have been observed [8, 48]. Accordingly, these guidelines stress the need for locally derived reference ranges for the management of pregnant women.

#### 3.2.8. Obesity

There appears to be a functional interrelationship between adiposity and thyroid status, in which TSH levels are correlated positively with body mass index [49]. TSH is reduced in obese patients who have undergone bariatric surgery, with little effect on T4 levels, consistent with a mechanism by which increased leptin levels drive increased TSH secretion in the setting of obesity [50]. Morbid obesity, in particular, leads to an isolated increase in serum TSH levels without any role for thyroid autoimmunity [49].

#### 3.2.9. Methodological Issues Relating to the Calculation of Reference Ranges

Recruiting a truly “normal” population for defining a reference range for TSH is challenging. Evidence from the US National Health and Nutrition Examination Survey (NHANES) found that about 4% of a population of community-dwelling subjects who were nominally free of thyroid dysfunction had evidence of occult autoimmune thyroid disease that increased the median TSH level and the upper reference limit for TSH [24, 51]. In another study, the use of sonography to detect and exclude people with thyroid abnormalities from a “healthy” reference population led to a lower reference range compared with the original group before these exclusions [52]. Other data have suggested limited benefit from adding sonography to the screening procedure for a TSH reference population, however [52–55].

Such observations have led for calls for the upper reference limit for TSH to be reduced, based on an observation that >95% of truly euthyroid patients have TSH <2.5 mIU/L and people with higher values were likely to have Hashimoto's thyroiditis or other conditions that increase TSH [56]. An alternative argument accepts that a substantial proportion of people in the upper part of the TSH reference range will indeed have some form of occult thyroid disease but with a sufficiently benign prognosis that it is unnecessary to alter reference ranges to reclassify these by defining healthy people as thyroid patients [57]. Guidance from the UK National Institute for Health and Care Excellence (NICE) recommends controlling TSH to the lower half of the reference range (0.4–2.5 mIU/L) for most people with subclinical hypothyroidism who require treatment [8].

Mathematical manipulation of TSH levels from a healthy TSH reference population is needed to define the TSH reference range, and precisely how this is done can lead to differences in the calculated reference values that are large enough to be clinically significant [58]. Data from 271 healthy control subjects and 820 patients with various forms of thyroid disease showed that different statistical methods used for derivation of the reference range could result in different classifications of the thyroid status for as many as 12% of the patients [59].

#### 3.2.10. Smoking

A recent analysis from the Korean National Health and Examination Survey evaluated associations between urinary cotinine (a metabolite of nicotine used routinely to assess exposure to tobacco) and thyroid function [60]. There was no significant association between FT4 and urinary cotinine, but TSH was correlated negatively with the cotinine levels in men and women, and levels of anti-TPO antibodies correlated positively with cotinine in men. These data confirm earlier reports of reduced TSH levels in smokers vs. nonsmokers [16].

#### 3.2.11. Variation in TSH Levels over Time

The diagnosis of subclinical hypothyroidism is categorical, as described above, so that small variations around the upper reference range for TSH can contribute to confirming or excluding the diagnosis of this condition. It is important to note that small increases in TSH above a stated reference range may resolve spontaneously. This was observed in an observational study where 37/40 people with subclinical hypothyroidism (defined as TSH >5 mIU/L) reverted to normal TSH level (by this definition) over a follow-up period of 5 years, with most reverting to a TSH level below this cutoff early in the follow-up period [61].

#### 3.2.12. Endocrine Disruptors

A growing literature is associating accumulation in the biosphere of a range of chemicals with alterations in numerous physiological functions, including the thyroid gland. Endocrine disruptors shown to influence thyroid function include a number of chemicals that are widely distributed in the environment, such as industrial chemicals used in the manufacture of plastics, flame retardants, fertilisers, and pesticides, among others [62]. The results of studies of endocrine disruptors on thyroid function have been conflicting to date, and thyroid hormone levels can be increased or decreased by exposure to these substances [62]. Importantly, early exposure to these substances may promote permanent changes in the regulation of the thyroid system [63, 64]. A recent commentary concluded that we are not ready to develop guidelines in this area, due to a lack of consistently designed studies, interregional and intersubject variability in the effects observed, and difficulty in estimating exposure [62]. The ubiquity of potential endocrine disruptors in the environment, and growing evidence of harm associated with them, identifies this area as important for the future in multiple fields of medicine.

## 4. Population Screening for Thyroid Dysfunction: We Can, but Should We?

Neonatal screening for congenital hypothyroidism (based on the measurement of TSH levels) is practised in many countries and is supported by guidelines [65]. There is less of a consensus regarding screening of the population for undiagnosed thyroid disease, which may be common among the general population. For example, about 6% of subjects in the NHANES III cohort in the USA had evidence of undiagnosed thyroid disease [24]. A meta-analysis of studies from Europe found a high prevalence of undiagnosed hypothyroidism of about 5% and of undiagnosed hyperthyroidism of about 2% [5]. Similarly, previously undiagnosed thyroid disease was found in an iodine-deficient area of Germany ten years following the introduction of iodine supplementation (subclinical hypothyroidism 0.5%; overt hypothyroidism 0.7%; subclinical hyperthyroidism 1.8%; overt hyperthyroidism 0.4%) [4].

Current TSH tests are reliable, automated, and suitable for routine use, provided that clinicians are aware of their limitations. The universal availability of routine TSH testing could render screening for primary thyroid disease, a realistic possibility. The introduction of population screening would require good evidence of the benefits and harms that would be expected to arise from the programme. A systematic review of studies that evaluated screening for the thyroid disease in the primary care setting in 1998 concluded that opportunistic screening of women aged >50 years may be beneficial but again called for large, randomised trials to validate the use of population screening for subclinical hypothyroidism [66]. Another systematic review of studies in this area (2003) concluded that the case for population screening for thyroid disorders was weak, when assessed against the current evidence base [67]. The current (2015) position of the US government concluded that there was still insufficient evidence to support population screening for thyroid dysfunction in nonpregnant, asymptomatic adults [68].

Population screening for thyroid disease remains an intriguing proposition. Randomised controlled trials to clarify optimal strategies for screening and intervention may be required before it becomes a practicable proposition, especially for detecting undiagnosed subclinical hypothyroidism.

## 5. Conclusions

TSH is the primary biomarker for the diagnosis of thyroid dysfunction and for guiding treatment of thyroid disease. The provision of clear and unambiguous laboratory reference ranges for “normal” TSH conceals a number of important caveats that should be remembered when using a TSH test for diagnosis. For the diagnosis of hypothyroidism, the upper cutoff for TSH that best predicts prognosis remains uncertain, and multiple demographic, laboratory, and disease characteristics interfere with the TSH test. It is important to manage the whole patient, considering these limitations, rather than treating solely on the basis of a slightly elevated TSH test result. In future, individually based TSH reference ranges, or optimal TSH levels based on prediction of adverse cardiovascular outcomes, may be useful in identifying many people with undiagnosed thyroid disease, although we do not have a sufficient evidence base for this approach at present. Furthermore, there is a pressing need to identify easily measurable biomarkers of tissue thyroid hormone status.
